# Estimation of Additive, Dominance, and Imprinting Genetic Variance Using Genomic Data

**DOI:** 10.1534/g3.115.019513

**Published:** 2015-10-04

**Authors:** Marcos S. Lopes, John W. M. Bastiaansen, Luc Janss, Egbert F. Knol, Henk Bovenhuis

**Affiliations:** * Topigs Norsvin Research Center, 6641SZ Beuningen, The Netherlands; † Animal Breeding and Genomics Centre, Wageningen University, 6708PB Wageningen, The Netherlands; ‡ Centre for Quantitative Genetics and Genomics, Aarhus University, DK-8830 Tjele, Denmark

**Keywords:** SNP, variance component, phenotype prediction, pigs, GenPred, shared data resource

## Abstract

Traditionally, exploration of genetic variance in humans, plants, and livestock species has been limited mostly to the use of additive effects estimated using pedigree data. However, with the development of dense panels of single-nucleotide polymorphisms (SNPs), the exploration of genetic variation of complex traits is moving from quantifying the resemblance between family members to the dissection of genetic variation at individual loci. With SNPs, we were able to quantify the contribution of additive, dominance, and imprinting variance to the total genetic variance by using a SNP regression method. The method was validated in simulated data and applied to three traits (number of teats, backfat, and lifetime daily gain) in three purebred pig populations. In simulated data, the estimates of additive, dominance, and imprinting variance were very close to the simulated values. In real data, dominance effects account for a substantial proportion of the total genetic variance (up to 44%) for these traits in these populations. The contribution of imprinting to the total phenotypic variance of the evaluated traits was relatively small (1–3%). Our results indicate a strong relationship between additive variance explained per chromosome and chromosome length, which has been described previously for other traits in other species. We also show that a similar linear relationship exists for dominance and imprinting variance. These novel results improve our understanding of the genetic architecture of the evaluated traits and shows promise to apply the SNP regression method to other traits and species, including human diseases.

Traditionally, exploration of genetic variance in humans, plants, and livestock species has been limited mostly to the use of additive effects estimated using pedigree data. In this context, the role of genetics in complex traits has been quantified as heritability, *e.g.*, the proportion of the total phenotypic variance explained by additive genetic variance (Visscher and Goddard 2014). However, the estimation of heritability via the use of additive models does not only capture additive gene action but can potentially also capture part of the dominance effects and epistatic interactions (Hill *et al.* 2008; Falconer and Mackay 1996). In addition, traditional additive models ignore imprinting effects, which also are expected to contribute to the genetic architecture and evolution of complex traits (Lawson *et al.* 2013; Cheng *et al.* 2013). Therefore, the proportion of phenotypic variation that is explained by all genetic effects and how much of the total genetic variation is actually due to additive effects is still unclear in modern genetics (Vinkhuyzen *et al.* 2013).

One of the main limitations to better understanding the genetic architecture of complex traits is that typically the data structure does not allow simultaneous estimation of additive, dominance, and imprinting variance (De Vries *et al.* 1994; Vitezica *et al.* 2013). Further, imprinting effects might be confounded with common litter or maternal effects (Wolf and Cheverud 2012; Tier and Meyer 2004). With the development of dense panels of single-nucleotide polymorphisms (SNPs), the exploration of genetic variation of complex traits is moving from the quantification of the resemblance between family members to the dissection of genetic variation at individual loci (Vinkhuyzen *et al.* 2013). Because these genetic effects can be estimated, simultaneously, we can now aim to quantify the contribution of additive, dominance, and imprinting variance to the total genetic variance.

Dominance effects are of great interest for both plant and livestock breeding because dominance has been suggested as one of the genetic mechanisms explaining heterosis (Visscher *et al.* 2000; Xiao *et al.* 1995; Davenport 1908; Bruce 1910; Shull 1908; Charlesworth and Willis 2009; Shen *et al.* 2014). Only recently, however, with the development of molecular genetics, attempts have been made to quantify and exploit the proportion of genetic variance due to dominance effects in plants (Muñoz *et al.* 2014) and livestock (Toro and Varona 2010; Su *et al.* 2012; Da *et al.* 2014; Nishio and Satoh 2014; Vitezica *et al.* 2013; Zeng *et al.* 2013; Sun *et al.* 2014). Regarding imprinting, hundreds of imprinted genes (*e.g.*, *IGF*2, *DIO*3, and *NOEY*2) have been identified in mammals (http://geneimprint.com/site/genes-by-species); however, the fraction of the total genetic variance due to imprinting effects has not yet been investigated using genomic data.

Moving beyond additive effects, *e.g.*, accounting for dominance and imprinting effects in addition to additive effects, may not only improve our understanding of the genetic architecture of complex traits, but also improve the prediction of phenotypes (de los Campos *et al.* 2010; Lee *et al.* 2008). This could be beneficial, for example, in predicting disease risk in humans (Wray *et al.* 2007) or for establishing mating strategies in plant or animal breeding aimed at maximizing the phenotypic performance of the (crossbred) offspring (Muñoz *et al.* 2014; Toro and Varona 2010).

The objective of this study was to estimate the contribution of additive, dominance, and imprinting effects to the total genetic variation with an SNP regression approach. The method was validated in simulated data and applied to three traits in three purebred pig populations.

## Materials and Methods

### Genomic data

A total of 2013 Landrace, 2402 Large White, and 1384 Pietrain animals were genotyped with the Illumina Porcine SNP60 Beadchip (Ramos *et al.* 2009). SNPs with call rate <0.95, minor allele frequency <0.05, strong deviation from Hardy-Weinberg equilibrium (χ^2^ > 600), GenCall < 0.15, unmapped SNPs, and SNPs located on sex chromosomes, according to the Sscrofa10.2 assembly of the reference genome (Groenen *et al.* 2012), were excluded from the data set. After quality control, 34,912 SNPs for Landrace, 36,578 SNPs for Large White, and 38,116 SNPs for Pietrain of the initial 64,232 SNPs were kept for phasing procedures. All animals had a frequency of missing genotypes <0.05; therefore, no animals were excluded due to high frequency of missing genotypes.

Phasing and imputation of missing genotypes were performed within each line via AlphaImpute (Hickey *et al.* 2011), which combines genomic and pedigree information to determine the parental origin of alleles. The pedigree depth used in this analysis was up to five generations (between genotyped animals).

For each SNP of each individual, AlphaImpute (Hickey *et al.* 2011) generates two probabilities, with P_1_ being the probability that a specific allele was received from its father, say an allele G of a G/C SNP, and P_2_ the probability that the same allele was received from its mother. Considering a heterozygous animal (GC) where the G allele was inherited, with certainty, from its father (and therefore a C allele from its mother), the probabilities would be P_1_= 1 and P_2_= 0. To obtain the regressors that allow the estimation of additive (*regA*), dominance (*regD*), and imprinting (*regI*) genetic variance, the following transformation of these probabilities was applied: *regA* = [(P_1_ + P_2_) – 1], *regD* = (|P_1_ – P_2_|), and *regI* = (P_1_ – P_2_). Thus, the genotypes (GG, GC, CG, CC) were recoded as (−1, 0, 0, 1), (0, 1, 1, 0) and (0, −1, 1, 0) to evaluate additive, dominance and imprinting variances, respectively.

To ensure accurate phasing, only animals that had both parents or at least one parent and one sib genotyped were used in further steps. As the result of these restrictions, 1538 Landrace, 1595 Large White, and 1272 Pietrain animals were available for the estimation of variance components.

### Simulation

To verify whether our data structure and statistical model allow disentangling additive, dominance, and imprinting effects, we simulated for the Landrace population a trait with additive, dominance, and imprinting effects, with mean 0, total genetic variance equal to 0.30, and total phenotypic variance equal to 1. Real genotypes of this population were used in the simulation procedures. A total of 15 SNPs with minor allele frequency between 0.45 and 0.50 were selected randomly to have an effect on the trait [quantitative trait loci (QTL)]: five with only additive effects, five with only dominance effects, and five with only imprinting effects. These SNPs were located on different chromosomes that also were selected randomly. Each QTL accounted for 2% of the total phenotypic variance. Therefore, the additive, dominance, and imprinting heritabilities were 10% each. The genetic variance (V_G_) of a single QTL was defined as described by De Koning *et al.* (2002):

$$\begin{array}{c} {\text{V}}_{\text{G}}={\text{V}}_{\text{a}}+{\text{V}}_{\text{d}}+{\text{V}}_{\text{i}}\\ {\text{V}}_{\text{a}}=2pq\;{\left[a+d\left(p-q\right)\right]}^{2}\\ {\text{V}}_{\text{d}}={\left(2pqd\right)}^{2}\\ {\text{V}}_{\text{i}}=2pqi^{2} \end{array}$$

where V_a_, V_d_, and V_i_ are, respectively, the additive, dominance, and imprinting variances and *a*, *d*, and *i* are, respectively, the additive, dominance, and imprinting effects of a given QTL with allele frequencies *p* and *q*. Because each QTL was simulated to either have an additive, dominance, or imprinting effect, *a* of each additive QTL, *d* of each dominant QTL and *i* of each imprinted QTL could be defined as: $a=\sqrt{\frac{0.02}{2pq}}$, $d=\frac{\sqrt{0.02}}{2pq}$ and $i=\sqrt{\frac{0.02}{2pq}}$. The simulated phenotype of the *j*^th^ animal then becomes:

$$phenotype_{j}=\sum_{s=1}^{n=15}(regA_{js}a_{s}+regD_{js}d_{s}+regI_{js}i_{s})+e_{j}$$

where *n* is the number of QTL (SNPs) affecting the trait, *e* is a random environmental component sampled from a random distribution with variance equal to 0.70, and *regA*, *regD*, and *regI* are defined as described in the section *Genomic data*. We generated 10 replicates of this simulation, and SNPs that met the selection criteria were allowed to have an effect in only one of the replicates.

### Phenotypes

The phenotypic data consisted of the traits number of teats, backfat, and lifetime daily gain, which corresponds to the average daily weight increase from birth to ∼120 kg. The response variables used to estimate the genetic variances were phenotypes preadjusted for fixed effects instead of the original observations. The preadjustment was based on a larger data set that included all contemporaneous animals of the genotyped animals, rather than just using the group of genotyped animals. Using this larger data set allowed us to account more accurately for contemporary group effects. The fixed effects estimates used for the preadjustment of the phenotypes were obtained by fitting a single trait pedigree-based linear model using ASReml v3.0 (Gilmour *et al.* 2009). The model for number of teats consisted of sex and herd-year-season as fixed effects and an additive genetic effect and a residual as random effects. The model for backfat consisted of sex, herd-year-week, and weight as fixed effects and an additive genetic effect, common litter effect, and a residual as random effects. For lifetime daily gain, the model consisted of sex and herd-year-week as fixed effects and an additive genetic effect, common litter effect and a residual as random effects. For the Landrace population, the final data set consisted of 141,248 animals for number of teats, 36,413 animals for backfat, and 37,071 animals for lifetime daily gain. For the Large White population, the final data set consisted of 156,065 animals for number of teats, 41,192 animals for backfat, and 41,740 animals for lifetime daily gain. For the Pietrain population, the final data set consisted of 33,964 animals for backfat and 31,184 animals for lifetime daily gain. The trait number of teats was not recorded in the Pietrain population. Descriptive statistics of the phenotypes are shown in Table 1.

**Table 1:** Descriptive statistics

Dataset	NT, units			BF, mm			DG, g		
	N	μ	SD	N	μ	SD	N	μ	SD
Landrace									
All	141,248	15.27	1.06	36,413	12.47	2.54	37,071	598.47	70.63
Genotyped	1538	15.62	1.04	1405	12.55	2.20	1394	628.27	62.93
Large White									
All	156,065	15,08	1.05	41,192	12,38	2.49	41,740	632.30	71.78
Genotyped	1595	15.40	0.98	1453	12.20	2.37	1468	649.86	69.09
Pietrain									
All				33,964	7.98	1.49	31,184	603.86	75.89
Genotyped				1272	7.82	1.28	1145	630.70	65.64

Number of animals with phenotypic information (N), mean (μ), and standard deviation (SD) of the traits number of teats (NT), backfat (BF), and lifetime daily gain from birth to ∼120 kg (DG).

### Statistical analyses

Parameters were estimated using models with random regression on SNP genotypes. Single trait within-line analyses were performed with three different models implemented in the program BayZ (http://www.bayz.biz/), the same for both real and simulated data:

$$\begin{array}{l} y=1\text{µ}+\left(\mathrm{Lb}\right)+\mathrm{Aa}+e\;\left(\text{MA model}\right)\\ y=1\text{µ}+\left(\mathrm{Lb}\right)+\mathrm{Aa}+\mathrm{Dd}+e\;\left(\text{MAD model}\right)\\ y=1\text{µ}+\left(\mathrm{Lb}\right)+\mathrm{Aa}+\mathrm{Dd}+\mathrm{Ii}+e\;\left(\text{MADI model}\right) \end{array}$$

where **y** is a vector of preadjusted phenotypic observations; *µ* is the mean of the populations and **1** a vector of ones; **L** is the design matrix for the common litter effects (only used for backfat and lifetime daily gain); **b** is a unknown vector of common litter effects; **A**, **D**, and **I** are design matrices with regressors for additive, dominance, and imprinting effects respectively; **a**, **d,** and **i** are unknown vectors of additive, dominance, and imprinting effects, respectively;, and **e** is a vector of residuals. The entries of the design matrices **A**, **D,** and **I** are regressors calculated from the observed phased probabilities of the marker genotypes (*regA*, *regD*, and *regI*), as described in the genomic data section above.

Assumed distributions were: **a** ∼ *N*(**0**,**I$\sigma_{a}^{2}$**), **d** ∼ *N*(**0**,**I$\sigma_{d}^{2}$**), **i** ∼ *N*(**0**,**I$\sigma_{i}^{2}$**), **b** ∼ *N*(**0**,**I$\sigma_{\text{L}}^{2}$**), and **e** ∼ *N*(**0**,**I$\;\sigma_{e}^{2}$**), with $\sigma_{a}^{2}$, $\sigma_{d}^{2}$, $\sigma_{i}^{2}$ being the per-SNP variance for additive, dominance, and imprinting effects, and $\sigma_{L}^{2}$ and $\sigma_{e}^{2}$ the common litter and residual variance, respectively. The model was fitted with a Bayesian approach in the Bayz software package (http://www.bayz.biz/) as described by Krag *et al.* (2013) to estimate variance components and heritabilities in SNP-based models. The prior distributions for unknown variance parameters were set as unbounded uniform, which makes the Bayesian posterior distribution mathematically identical to the likelihood. The generated Monte Carlo chain starts with all regression parameters and other location parameters at zero, and all variance parameters at 1, and blocked Gibbs samplers are used to facilitate mixing. Each model was run as a single chain with a length of 500,000 (real data) and 100,000 (simulated data), which was sampled each 100 iterations. The first 50,000 iterations of each run were regarded as burn-in period.

Because the evaluated models were SNP-based models, they do not readily provide estimates of total explained variance. One way to obtain the total explained variance from a model term like **Aa** is to write *var*(**Aa**) = **AA**′$\sigma_{a}^{2}$ and compute or evaluate the expected average diagonal of **AA**′ to provide the constant to scale the per-SNP explained variance to total explained variance. In this way total variance in the models can be expressed as $[\left(\sigma_{\text{L}}^{2}\right)+\sigma_{\mathrm{Aa}}^{2}$ + $\sigma_{e}^{2}]$ for MA, $[\left(\sigma_{\text{L}}^{2}\right)+\sigma_{\mathrm{Aa}}^{2}$ + $\sigma_{\mathrm{Dd}}^{2}$ + $\sigma_{e}^{2}]$ for MAD and $[\left(\sigma_{\text{L}}^{2}\right)+\sigma_{\mathrm{Aa}}^{2}$ + $\sigma_{\mathrm{Dd}}^{2}$ + $\sigma_{Ii}^{2}$ + $\sigma_{e}^{2}]$ for MADI, with $\sigma_{\mathrm{Aa}}^{2}$= **AA’$\sigma_{a}^{2}$** (total additive variance), $\sigma_{Dd}^{2}$= **DD’$\sigma_{d}^{2}$** (total dominance variance), and $\sigma_{Ii}^{2}$= **II’$\sigma_{i}^{2}$** (total imprinting variance). Only for backfat and lifetime daily gain, $\sigma_{\text{L}}^{2}$ was included. Alternatively, the variance contributed by a random effect could be estimated by evaluating the sample variance of the entries of the vectors **Aa, Dd**, and **Ii** at each iteration of the Gibbs sampler (Sorensen *et al.* 2001). This has the advantage that the posterior standard deviations also can be obtained for total explained variance, and explained variances can be split easily by chromosome. The latter is done by computing var(**Aa**) per Markov chain Monte Carlo cycle only for the part of the covariates in **A** and matching regression parameters in **a** that belong to a particular chromosome. The narrow-sense heritability was defined as $\sigma_{Aa}^{2}$/$\sigma_{\text{P}}^{2}$, and the proportion of phenotypic variance explained by dominance and imprinting effects was defined as $\sigma_{Dd}^{2}$/$\sigma_{\text{P}}^{2}$ and $\sigma_{Ii}^{2}$/$\sigma_{\text{P}}^{2}$, respectively.

The portioning of the genetic variance as described previously has been defined as the “genotypic model” (Vitezica *et al.* 2013), which implies that $\sigma_{Aa}^{2}$, $\sigma_{Dd}^{2}$, and $\sigma_{Ii}^{2}$ are the variance of the genotypic additive, dominance, and imprinting values, respectively. The genotypic model and the breeding (or classical) model are statistically equivalent (*i.e.*, they lead to the same probability model). However, the parameters obtained with these models have different interpretations. In the genotypic model, the additive variance is the variance of additive effects (average difference between homozygotes), whereas in the breeding model, additive effects are functions of allele substitution effects. To make the variance estimates from the genotypic model comparable with the estimates of the breeding model, a transformation of these results was proposed by Vitezica *et al.* (2013). We have applied the transformation proposed by Vitezica *et al.* (2013) to the estimates from the MAD model and included the results in the supporting material (supporting information, File S1).

Finally, we evaluated whether the proportion of variance explained by a single chromosome was related to its physical length. The length of a given chromosome was defined as the distance between the first and the last SNP on this chromosome according to the Sscrofa 10.2 assembly (Groenen *et al.* 2012). The relationship between variance explained and physical length of the chromosome was expressed as the coefficient of determination (r^2^) from the regression of variance explained on physical length. Variance explained by each chromosome individually was obtained based on the effects of SNPs on that chromosome. SNP effects were from the analyses where all SNPs from all chromosomes were evaluated simultaneously. Variance per chromosome was estimated for all three models evaluated (MA, MAD, and MADI).

### Model comparison

Models were compared using the Deviance Information Criterion (DIC, Spiegelhalter *et al.* 2002). DIC is widely used for Bayesian model comparison and is analogous to the Akaike Information Criterion (Akaike 1974). DIC combines a measure of model fit (the expected deviance) with a measure of model complexity (the effective number of parameters) over all iterations after burn-in. The model with lowest DIC is chosen as the best fitting model (Spiegelhalter *et al.* 2002).

### Data availability

Relevant data is available as supplemental information.

## Results

### Simulation

Average narrow-sense heritability over the 10 replicates of the simulated trait was estimated at 0.116, 0.092, and 0.098 using the MA, MAD, and MADI models, respectively (Table 2). The average proportion of phenotypic variance explained by dominance effects was 0.111 using the MAD model and 0.097 using the MADI model. Using the MADI model, the average proportion of phenotypic variance explained by imprinting effects was 0.103.

**Table 2:** Estimated variance components and proportion of phenotypic variance ($\sigma_{\text{P}}^{2}$) explained by additive, dominance, and imprinting effects for the simulated data

Model	Variance Components				Variance Explained		
	$\sigma_{e}^{2}$	$\sigma_{Aa}^{2}$	$\sigma_{Dd}^{2}$	$\sigma_{Ii}^{2}$	$\sigma_{Aa}^{2}$/$\sigma_{\text{P}}^{2}$ *	$\sigma_{Dd}^{2}$/$\sigma_{\text{P}}^{2}$	$\sigma_{Ii}^{2}$/$\sigma_{\text{P}}^{2}$
MA	0.869 ± 0.035	0.115 ± 0.036			0.116 ± 0.035		
MAD	0.789 ± 0.038	0.091 ± 0.035	0.110 ± 0.048		0.092 ± 0.035	0.111 ± 0.047	
MADI	0.698 ± 0.041	0.097 ± 0.033	0.097 ± 0.057	0.102 ± 0.028	0.098 ± 0.027	0.097 ± 0.061	0.103 ± 0.032
Simulated	0.700	0.100	0.100	0.100	0.100	0.100	0.100

$\sigma_{e}^{2}$, residual variance; $\sigma_{Aa}^{2}$, total additive variance; $\sigma_{Dd}^{2}$, total dominance variance; $\sigma_{Ii}^{2}$, total imprinting variance. **$\sigma_{Aa}^{2}$**/$\sigma_{\text{P}}^{2}$*, narrow-sense heritability; MA, model including only additive effects; MAD, model including additive and dominance effects; MADI, model including additive, dominance, and imprinting effects.

The pairwise sampling correlation between additive, dominance, imprinting and error variance are shown in Table S1. The average correlation between the different variances of the 10 replicates of the simulated trait ranged from −0.586 to 0.018. The strongest correlations (−0.586 to −0.186) were observed between the residual variance and the variance of the three components of genetic variance evaluated. The lowest correlations (approximately zero) were observed between imprinting and additive variance and between imprinting and dominance variance.

### Real data

For the trait number of teats, the narrow-sense heritability estimated using MA was 0.319 in the Landrace and 0.343 in the Large White population (Table 3 and Table 4). When both MAD and MADI were used, the narrow-sense heritability was approximately the same in both populations (∼0.306). The estimates of the proportion of phenotypic variance explained by dominance effects, however, showed a large difference between populations. The proportion of phenotypic variance explained by dominance effects for number of teats in the Landrace population (0.039) was a little bit more than one third that of the Large White population (∼0.100). The proportion of phenotypic variance explained by imprinting effects for number of teats was low in both populations, 0.015 in the Landrace and 0.010 in the Large White.

**Table 3:** Variance components and proportion of phenotypic variance ($\sigma_{\text{P}}^{2}$) explained by additive, dominance, and imprinting effects for the real data in the Landrace population

Trait	Model	Variance Components					Variance Explained		
		$\sigma_{\text{e}}^{2}$	$\sigma_{\text{L}}^{2}$	$\sigma_{Aa}^{2}$	$\sigma_{Dd}^{2}$	$\sigma_{Ii}^{2}$	$\sigma_{Aa}^{2}$/$\sigma_{\text{P}}^{2}$ *	$\sigma_{Dd}^{2}$/$\sigma_{\text{P}}^{2}$	$\sigma_{Ii}^{2}$/$\sigma_{\text{P}}^{2}$
NT	MA	0.739 ± 0.042		0.346 ± 0.041			0.319 ± 0.035		
	MAD	0.714 ± 0.046		0.334 ± 0.045	0.042 ± 0.034		0.306 ± 0.037	0.039 ± 0.031	
	MADI	0.700 ± 0.046		0.334 ± 0.043	0.042 ± 0.036	0.016 ± 0.014	0.305 ± 0.036	0.039 ± 0.033	0.015 ± 0.012
BF	MA	1.232 ± 0.120	0.431 ± 0.115	1.803 ± 0.150			0.520 ± 0.035		
	MAD	1.082 ± 0.138	0.362 ± 0.117	1.604 ± 0.199	0.345 ± 0.172		0.472 ± 0.047	0.102 ± 0.052	
	MADI	1.031 ± 0.135	0.369 ± 0.119	1.596 ± 0.195	0.345 ± 0.166	0.057 ± 0.038	0.469 ± 0.046	0.102 ± 0.050	0.017 ± 0.011
DG	MA	1,435 ± 111	380 ± 106	662 ± 113			0.267 ± 0.043		
	MAD	1,213 ± 141	314 ± 105	525 ± 122	446 ± 185		0.210 ± 0.047	0.178 ± 0.071	
	MADI	1,185 ± 138	319 ± 104	554 ± 124	395 ± 179	47 ± 34	0.221 ± 0.048	0.158 ± 0.070	0.019 ± 0.014

$\sigma_{e}^{2}$, residual variance; $\sigma_{\text{L}}^{2}$, common litter variance; $\sigma_{Aa}^{2}$, total additive variance; $\sigma_{Dd}^{2}$, total dominance variance; $\sigma_{Ii}^{2}$, total imprinting variance; **$\sigma_{Aa}^{2}$**/$\sigma_{\text{P}}^{2}$*, narrow-sense heritability; NT, number of teats; BF, backfat; DG, average daily gain from birth to ∼120 kg; MA, model including only additive effects; MAD, model including additive and dominance effects; MADI, model including additive, dominance, and imprinting effects.

**Table 4:** Variance components and proportion of phenotypic variance ($\sigma_{\text{P}}^{2}$) explained by additive, dominance, and imprinting effects of the real data in the Large White population

Trait	Model	Variance Components					Variance Explained		
		$\sigma_{\text{e}}^{2}$	$\sigma_{\text{L}}^{2}$	$\sigma_{Aa}^{2}$	$\sigma_{Dd}^{2}$	$\sigma_{Ii}^{2}$	$\sigma_{Aa}^{2}$/$\sigma_{\text{P}}^{2}$ *	$\sigma_{Dd}^{2}$/$\sigma_{\text{P}}^{2}$	$\sigma_{Ii}^{2}$/$\sigma_{\text{P}}^{2}$
NT	MA	0.637 ± 0.032		0.333 ± 0.032			0.343 ± 0.029		
	MAD	0.578 ± 0.041		0.299 ± 0.037	0.094 ± 0.043		0.307 ± 0.034	0.097 ± 0.044	
	MADI	0.563 ± 0.040		0.300 ± 0.038	0.103 ± 0.043	0.010 ± 0.009	0.307 ± 0.035	0.105 ± 0.043	0.010 ± 0.009
BF	MA	1.086 ± 0.090	0.371 ± 0.084	0.931 ± 0.093			0.390 ± 0.034		
	MAD	0.927 ± 0.105	0.307 ± 0.085	0.848 ± 0.105	0.320 ± 0.125		0.353 ± 0.039	0.133 ± 0.051	
	MADI	0.834 ± 0.115	0.302 ± 0.084	0.852 ± 0.104	0.353 ± 0.134	0.071 ± 0.041	0.353 ± 0.039	0.146 ± 0.055	0.029 ± 0.017
DG	MA	1,867 ± 129	282 ± 114	682 ± 100			0.241 ± 0.033		
	MAD	1,715 ± 144	234 ± 108	590 ± 120	300 ± 157		0.208 ± 0.040	0.106 ± 0.055	
	MADI	1,659 ± 149	230 ± 108	557 ± 129	370 ± 178	34 ± 29	0.195 ± 0.043	0.130 ± 0.062	0.012 ± 0.010

$\sigma_{e}^{2}$, residual variance; $\sigma_{\text{L}}^{2}$, common litter variance; $\sigma_{Aa}^{2}$, total additive variance; $\sigma_{Dd}^{2}$, total dominance variance; $\sigma_{Ii}^{2}$, total imprinting variance; **$\sigma_{Aa}^{2}$**/$\sigma_{\text{P}}^{2}$*, narrow-sense heritability; NT, number of teats; BF, backfat; DG, average daily gain from birth to ∼120 kg; MA, model including only additive effects; MAD, model including additive and dominance effects; MADI, model including additive, dominance, and imprinting effects.

For the trait backfat, the narrow-sense heritability estimated using MA was 0.520, 0.390, and 0.419 in the Landrace, Large White, and Pietrain populations, respectively. Additive heritabilities decreased to 0.469, 0.353, and 0.394 in the Landrace, Large White, and Pietrain populations, respectively, when the MA model was replaced by MADI (Table 3, Table 4, and Table 5). MAD and MADI resulted in almost the same estimates of narrow-sense heritability, and of the proportion of phenotypic variance explained by dominance effects for backfat in all populations and narrow-sense heritability was always lower than the estimate based on MA. Similar to number of teats, the proportion of phenotypic variance explained by dominance effects for backfat was variable between populations with estimates of 0.102 in the Landrace, 0.146 in the Large White, and 0.064 in the Pietrain population. The proportion of phenotypic variance explained by imprinting effects for backfat was 0.017 in the Landrace, 0.029 in the Large White, and 0.020 in the Pietrain population.

**Table 5:** Variance components and proportion of phenotypic variance ($\sigma_{\text{P}}^{2}$) explained by additive, dominance, and imprinting effects for the real data in the Pietrain population

Trait	Model	Variance Components					Variance Explained		
		$\sigma_{\text{e}}^{2}$	$\sigma_{\text{L}}^{2}$	$\sigma_{Aa}^{2}$	$\sigma_{Dd}^{2}$	$\sigma_{Ii}^{2}$	$\sigma_{Aa}^{2}$/$\sigma_{\text{P}}^{2}$ *	$\sigma_{Dd}^{2}$/$\sigma_{\text{P}}^{2}$	$\sigma_{Ii}^{2}$/$\sigma_{\text{P}}^{2}$
BF	MA	0.610 ± 0.050	0.096 ± 0.041	0.510 ± 0.054			0.419 ± 0.038		
	MAD	0.570 ± 0.056	0.082 ± 0.040	0.476 ± 0.064	0.085 ± 0.060		0.392 ± 0.046	0.070 ± 0.050	
	MADI	0.551 ± 0.060	0.084 ± 0.041	0.481 ± 0.062	0.078 ± 0.058	0.024 ± 0.019	0.394 ± 0.044	0.064 ± 0.047	0.020 ± 0.016
DG	MA	1,804 ± 143	223 ± 123	931 ± 130			0.314 ± 0.040		
	MAD	1,488 ± 163	148 ± 102	735 ± 150	584 ± 195		0.248 ± 0.047	0.198 ± 0.065	
	MADI	1,468 ± 172	154 ± 101	718 ± 169	591 ± 239	32 ± 31	0.242 ± 0.054	0.199 ± 0.080	0.011 ± 0.010

$\sigma_{e}^{2}$, residual variance;$\;\sigma_{\text{L}}^{2}$, common litter variance; $\sigma_{Aa}^{2}$, total additive variance; $\sigma_{Dd}^{2}$, total dominance variance; $\sigma_{Ii}^{2}$, total imprinting variance; **$\sigma_{Aa}^{2}$**/$\sigma_{\text{P}}^{2}$*, narrow-sense heritability; BF, backfat; DG, average daily gain from birth to ∼120 kg; MA, model including only additive effects; MAD, model including additive and dominance effects; MADI, model including additive, dominance, and imprinting effects.

Finally, for the trait lifetime daily gain, the narrow-sense heritability estimated using MA was 0.267 in the Landrace, 0.241 in the Large White, and 0.314 in the Pietrain population (Table 3, Table 4, and Table 5). Again, the narrow-sense heritability and the proportion of phenotypic variance explained by dominance effects were similar with the use of either MAD or MADI in all populations, and additive estimates were smaller than those from the MA model. The proportion of phenotypic variance explained by dominance effects estimated with MADI were greater for lifetime daily gain than for the other two traits in the Landrace (0.158) and Pietrain populations (0.199) and similar to dominance for backfat in the Large White population (0.130). The proportion of phenotypic variance explained by imprinting effects of lifetime daily gain was again low, as for the other 2 traits (<0.020 in all populations).

### Model comparison

Based on the estimated DIC (Table 6), MAD and MADI presented a better fit to the data than MA for all traits in all populations, except for number of teats in the Landrace population (which was the trait with the lowest proportion of dominance variance in this study). The MADI model was slightly superior to MAD for backfat in the Landrace population, for all traits in the Large White population, and for lifetime daily gain in the Pietrain population.

**Table 6:** Deviance information criterion

Population	Trait	MA	MAD	MADI
Landrace	NT	**1418**	1431	1458
	BF	2199	2133	**2093**
	DG	11,433	**11,325**	11,333
Large White	NT	1225	1206	**1180**
	BF	2174	2051	**1992**
	DG	12,894	12,861	**12,838**
Pietrain	BF	1089	**1051**	1053
	DG	10,059	9954	**9952**

Numbers given in bold indicate the lowest deviance information criterion (best fit) obtained for each trait in each population. MA, model including only additive effects; MAD, model including additive and dominance effects; MADI, model including additive, dominance, and imprinting effects; NT, number of teats; BF, backfat; DG, average daily gain from birth to ∼120 kg.

### Variance explained by individual chromosomes

After estimation of SNP effects using all SNPs simultaneously, we estimated the additive, dominance, and imprinting variance by using the complete set of SNPs and also the variances per individual chromosomes. Estimates obtained from genome-wide SNPs were close to results from adding up the contributions of individual chromosomes. The largest difference was observed for number of teats in the Landrace population. The additive variance estimated using all SNPs was 0.33 for number of teats, while adding up the contributions of individual chromosomes resulted in an estimate of 0.39 for number of teats. The variance explained per chromosome for all traits in all populations using MADI is shown in File S2. We report results obtained using MADI. Very similar results were observed with all three models (MA, MAD, and MADI).

For all traits, the proportion of additive, dominance, and imprinting variance explained per chromosome showed a strong linear relationship with chromosome length (r^2^ ranging from 0.84 to 0.94).

## Discussion

### Simulation

Analysis of simulated data with the MADI model resulted in estimates of additive, dominance, and imprinting variance that were very close to the simulated values (Table 2). However, the additive variance was overestimated when using the MA model and the dominance variance was overestimated with the MAD model. This is a sensible result as all models, except the MADI model, are under-parameterized. A model that allows a proper dissection of the variances should yield variance components that are uncorrelated (Hill 2010). To test this we calculated the pairwise sampling correlations between the error, additive, dominance, and imprinting variance. The pairwise sampling correlations were moderate and mostly negative (Table S1). On the basis of these simulation results, we therefore concluded that data structure and the methodology will allow us to disentangle additive, dominance and imprinting variance, although this simulated scenario may not be representative of the genetic architecture of a real complex trait.

### Real data

In all populations, we observed a reduction in the narrow-sense heritability of all evaluated traits when dominance effects were accounted for (*e.g.*, using MAD instead of MA). The smallest decrease in narrow-sense heritability was observed for number of teats (4.2%) and the highest for lifetime daily gain (21.3%), both in the Landrace population (Table 3). The broad-sense heritability (sum of the heritabilities due to all genetic effects used in the model) of all evaluated traits increased in all three populations when dominance and imprinting effects were added to the model. The broad-sense heritability of lifetime daily gain was >30% greater when using MADI compared to using MA (Table 3, Table 4, and Table 5). A reduction of additive genetic variance and an increase in the broad-sense heritability was previously reported (Su *et al.* 2012) when nonadditive genetic effects were included in the model to evaluate daily gain in pigs. For height in trees, the narrow-sense heritability was found to reduce by 26% with the inclusion of nonadditive genetic effects in the model (Muñoz *et al.* 2014). According to Muñoz *et al.* (2014) and Pante *et al.* (2002), such a reduction in the additive genetic variance should be expected when dominance effects are present, since dominance effects also contribute to the additive genetic variance in the MA model. However, Vitezica *et al.* (2013) reported that such a reduction in the additive variance should be seen as an underestimation, as a consequence of overestimating the dominance variance. These authors described that when dominance is fitted in genotypic models (such as the one applied in the current study and by Su *et al.* (2012) and Muñoz *et al.* (2014), the part of the dominance effect that contributes to the allele substitution effect is shifted to the dominance variance. Because of this shift, the estimates from genotypic models are not directly comparable to pedigree-based estimates. Therefore, with methodology applied in the current study we could interpret the decrease in the narrow-sense heritability from the MA model compared to the MAD model as the contribution of dominance effects to the additive genetic variance. If the aim is to estimate breeding values and dominance deviations (*i.e.*, the traditional breeding model), the parameterization proposed by Vitezica *et al.* (2013) should be applied.

The substantial greater values for the broad-sense heritability compared to the narrow-sense heritability indicate that dominance effects make an important contribution to the genetic variance of the evaluated traits and populations in this study, especially to the trait lifetime daily gain. For lifetime daily gain in the Pietrain population using MADI, we observed the greatest proportion of phenotypic variance explained by dominance effects (0.198), with a ratio between dominance and additive variance of 0.82. In other lines, the ratios were also high. Using the same model, we found that the ratio between dominance and additive variance was 0.71 in the Landrace and 0.66 in the Large White population. Moreover, our results also show that additive variance accounts for the largest fraction of the genetic variance. This is in agreement with a previous study that described that additive variance is expected to account for >50% (often about 100%) of the total genetic variance (Hill *et al.* 2008). However, the estimates of additive variance of the traits in the populations here evaluated might still become smaller if epistatic interactions exist and would be included as a separate variance component. Although the role of epistatic interactions in the genetic architecture of complex traits has been investigated in different species (Muñoz *et al.* 2014; Su *et al.* 2012; Le Rouzic *et al.* 2008), we did not attempt to estimate epistatic variance because the power to identify these effects in segregating populations was expected to be low (Melchinger *et al.* 2007). To be able to detect epistatic interactions in outbred populations, loci with these effects should have a large effect and segregate with an intermediate frequency (Hill *et al.* 2008).

Individual loci that show the effect of imprinting have been identified for pigs, such as *IGF*2 (insulin-like growth factor 2 gene, Jeon *et al.* 1999; Nezer *et al.* 1999). The contribution of imprinting to the total genetic variance is, however, still unknown. No reports were found in literature that attempt to quantify total imprinting variance using genomic data. In this study, the trait with the greatest proportion of phenotypic variance explained by imprinting effects was backfat (0.017 in the Landrace, 0.029 in the Large White, and 0.020 in the Pietrain population), although the estimates were still quite low, in comparison with the amount of narrow-sense heritability and the proportion of phenotypic variance explained by dominance effects. In mice, a gene expression QTL mapping study for body composition traits showed that imprinting QTL accounted for only a limited amount of the phenotypic variance (<2.50%) for most traits (Cheng *et al.* 2013). In a pedigree-based study in pigs, it was shown that 5–7% of the phenotypic variance of backfat and 1–4% of growth rate was explained by paternal imprinting (De Vries *et al.* 1994). That study also showed maternal imprinting to account for 2–5% and 3–4% of the phenotypic variance of backfat and growth rate, respectively. Although our genomic estimates are lower than the pedigree-based estimates described by De Vries *et al.* (1994), the two results agree that imprinting effects are more important for backfat than for growth traits. The amount of phenotypic variance of number of teats due to imprinting effects has not yet been described in the literature. However, two imprinted QTL have been reported on chromosomes 2 and 12 (Hirooka *et al.* 2001). These two QTL explained 1.3 and 2.2% of the phenotypic variance of number of teats, while in our study the proportion of phenotypic variance explained by imprinting effects of number of teats in both populations was ≤1.5%. The larger imprinting variances found by Hirooka *et al.* (2001) may in part be explained by the design, an experimental F2 population, analyzed in their QTL study.

Due to the low proportion of phenotypic variance explained by imprinting, the relevance of estimating imprinting effects may be low when the aim is to predict the phenotypes number of teats, backfat, and lifetime daily gain in the evaluated populations. However, this study shows that, when present, dominance and imprinting variance can be detected and estimated with a SNP regression model. Using pedigree-based analysis this would typically not be feasible, for different reasons. First, the estimation of dominance variance using pedigree data requires data from large full-sib families (Vitezica *et al.* 2013), which is often not available in humans and livestock species. Second, pedigree-based methods have difficulties in disentangling imprinting from maternal and permanent environmental effects (Wolf and Cheverud 2012; Tier and Meyer 2004). Third, pedigree-based analysis often overestimates additive variance (Vinkhuyzen *et al.* 2013) and underestimates dominance variance (Muñoz *et al.* 2014). Although the use of genome-wide markers, compared to pedigree data, has been described as a more precise alternative to partition the genetic variance (Vinkhuyzen *et al.* 2013; Muñoz *et al.* 2014; Lee *et al.* 2008), it also has its pitfalls. If the causal variants are not in linkage disequilibrium with the SNPs used for the estimation of the variance components, their contribution to the variance will not be captured. The proportion of the variance explained by the SNPs is therefore likely to be underestimated (Vinkhuyzen *et al.* 2013). This phenomenon has been described as “the case of the missing heritability” (Maher 2008). Our genomic estimates of the additive genetic variance (Table 3, Table 4, and Table 5) were on average 28% lower than pedigree-based estimates that were obtained using the same data accounting only for additive effects. The pedigree-based heritability of number of teats was 0.340 in the Landrace and 0.420 in the Large White population; the pedigree-based heritability of backfat was 0.668 in the Landrace, 0.490 in the Large White and 0.513 in the Pietrain population; and the pedigree-based heritability of lifetime daily gain was 0.401 in the Landrace, 0.300 in the Large White and 0.474 in the Pietrain population (data not shown). Although these differences between the genomic and pedigree estimates are considerable, it is difficult to say if they are more likely due to an overestimation with pedigree or due to an underestimation with genomics. Nevertheless, using genomic data to estimate additive, dominance, and imprinting variances allows us to not only better understand the genetic architecture of the evaluated traits, but it might also improve the prediction of phenotypes compared to pedigree-based methods.

In recent studies, the inclusion of dominance effects in genomic evaluations of livestock has been reported to increase the accuracy and decrease the bias of estimated breeding values (Toro and Varona 2010; Su *et al.* 2012). In addition, using dominance in genomic evaluations is expected to result in greater cumulative response to selection of purebred animals for crossbred performance than additive models, especially in the presence of overdominance and when retraining is not performed at each generation (Zeng *et al.* 2013). Even when purely additive effects were evaluated, the inclusion of dominance in the genomic evaluations did not decrease the accuracy of prediction (Toro and Varona 2010; Su *et al.* 2012; Zeng *et al.* 2013). In plants, simultaneously accounting for additive and non-additive effects was more stable and yielded higher predictive ability of the mean phenotype than models that only account for additive effects (Muñoz *et al.* 2014). Also in mice, the prediction of phenotypes of complex traits using a model with additive and dominance effects has proven to be feasible and accurate (Lee *et al.* 2008). Therefore, combining additive, dominance, and imprinting under a genomic prediction scope opens new perspectives for the optimization of animal and plant breeding programs aiming for an improved prediction of crossbred performance, and also for identification of individuals that are at a risk for a given disease.

### Variance explained per individual chromosome

The strong linear relationship between chromosome length and proportion of variance explained per chromosome in our study was in line with the strong relationship between additive variance explained per chromosome and chromosome length previously described in humans (Yang *et al.* 2011) and in chickens (Abdollahi‐Arpanahi *et al.* 2014). Here we also showed that the same applies for dominance and imprinting variance. This indicates that the additive, dominance, and imprinting variance of number of teats, backfat and lifetime daily gain in these populations is explained by many genes located throughout the genome, rather than by a few mutations with large effects.

**Figure 1:**
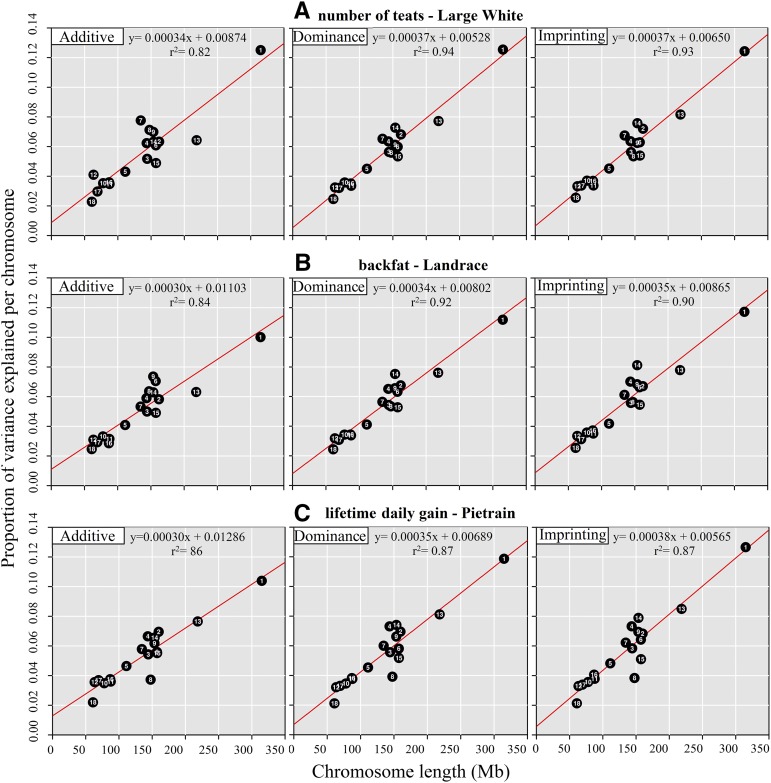
Proportion of additive, dominance, and imprinting variance explained per individual chromosome against physical length of the chromosome. (A) Number of teats in the Large White population; (B) backfat in the Landrace population; (C) lifetime daily gain in the Pietrain population.

The relationship between variance explained and chromosome length for number of teats in the Large White population, backfat in the Landrace population, and lifetime daily gain in the Pietrain population using MADI is illustrated in Figure 1. Although our results show that the variance of all three genetic effects have a strong relationship with chromosome length, the r^2^ for the additive variance was lower than the r^2^ for dominance and imprinting variance, especially in the Landrace and Large White populations. In addition, in the Pietrain population, the r^2^ for dominance and imprinting variance (Figure 1C) was lower than the r^2^ observed in the Landrace and Large White populations. This was observed because the proportion of variance explained by chromosome 8 in the Pietrain is clearly lower than in the Landrace and Large White populations. Having a closer look at the data of chromosome 8, we observed that the number of SNPs on this chromosome in the Pietrain population was on average 15% lower than in the Landrace and the Large White populations (n = 1632 in Pietrain, n = 1871 in Landrace, n = 1985 in Large White). Besides chromosome 8, the number of SNPs per chromosome was similar in all three populations. This difference in number of SNPs on chromosome 8 is observed because in the Pietrain population, compared to the Landrace and the Large White populations, more SNPs presented low minor allele frequency or were completely fixed and therefore were excluded from the estimation of the variance components. This large number of SNPs with low minor allele frequency (or completely fixed) could be due to an ascertainment bias due to the selection of SNPs for the SNP chip used in this study. However, this could be also an indication that genes that influence traits included in the selection index of Pietrain are located on this chromosome. The breeding objectives in Pietrain (sire line) are distinct from those in the Landrace and Large White (dam lines) which are more similar. Given this difference, some alleles could have moved to fixation in Pietrain but not in Landrace and Large White.

The proportion of additive variance explained by chromosome 7 for number of teats in the Large White population is relatively high (Figure 1A). This chromosome explained 21% more additive variance than chromosome 13, which is 62% longer than chromosome 7. This large proportion of explained variance is in agreement with the presence of a QTL for number of teats on this chromosome. In a previous study on a subset of the current Large White population, it was shown that on chromosome 7 a QTL is located in the region of the *VRTN* gene, explaining 2.5% of genetic variance (Duijvesteijn *et al.* 2014). In the current study, we showed that chromosome 7 accounted for 7.75% of the additive variance (5.64% of the total genetic variance using MADI).

Dominance effects account for a large proportion of the total genetic variance (up to 44%) for number of teats, backfat and lifetime daily gain in the pig populations evaluated. Although the contribution of imprinting effects to the total phenotypic variance of the evaluated traits was relatively small (1–3%), the SNP regression method allowed estimation of the additive, dominance and imprinting effects and resulting variances. Our results indicate a strong relationship between additive variance explained per chromosome and chromosome length, which has been previously described for other traits in other species. In addition, we also show that a similar linear relationship exists for dominance and imprinting variance. These novel results improve our understanding of the genetic architecture of the evaluated traits. The model can now be applied to other traits and species. Our results also open new perspectives for the inclusion of dominance and imprinting effects in prediction of phenotypes, especially regarding mate allocation techniques in animal and plant breeding, and for assessment of the risk of disease in humans.

## Supplementary Material

Supporting Information
