# Supplementary material for: Estimation of Additive, Dominance, and Imprinting Genetic Variance Using Genomic Data
Source: G3 (Bethesda). 2015 Oct 4;5(12):2629–37. doi: 10.1534/g3.115.019513 (PMC4683636; doi:10.1534/g3.115.019513)
Supplement: Supporting Information [file supp_5_12_2629__index.html]

Estimation of Additive, Dominance, and Imprinting Genetic Variance Using Genomic Data — Supporting Information 

# Estimation of Additive, Dominance, and Imprinting Genetic Variance Using Genomic Data

## Supporting Information for Lopes et al., 2015

**Files in this Data Supplement:**

- Table S1 - Sampling correlation between variance estimates of the simulated trait (.docx, 23 KB)
- File S1 - Variance components estimated using the model MAD transformed to the breeding model as proposed by Vitezica et al. (.docx, 27 KB)
- File S2 - Variance explained per chromosome (.xlsx, 26 KB)
