# Supplementary material for: Estimation of Additive, Dominance, and Imprinting Genetic Variance Using Genomic Data
Source: G3 (Bethesda). 2015 Oct 4;5(12):2629–37. doi: 10.1534/g3.115.019513 (PMC4683636; doi:10.1534/g3.115.019513)
Supplement: Supporting Information [file supp_g3.115.019513_TableS1.docx]

**Table S1 Sampling correlation between variance estimates of the simulated trait**

| **Model** |  | $\sigma_{e}^{2}$ | $\sigma_{\mathbf{Aa}}^{2}$ | $\sigma_{\mathbf{Dd}}^{2}$ |
| --- | --- | --- | --- | --- |
| **MA** | $\sigma_{\mathbf{Aa}}^{2}$ | -0.504 |  |  |
| **MAD** | $\sigma_{\mathbf{Aa}}^{2}$ | -0.186 |  |  |
|  | $\sigma_{\mathbf{Dd}}^{2}$ | -0.586 | -0.208 |  |
| **MADI** | $\sigma_{\mathbf{Aa}}^{2}$ | -0.204 |  |  |
|  | $\sigma_{\mathbf{Dd}}^{2}$ | -0.505 | -0.215 |  |
|  | $\sigma_{\mathbf{Ii}}^{2}$ | -0.422 | -0.006 | 0.018 |
