# Supplementary material for: Estimation of Additive, Dominance, and Imprinting Genetic Variance Using Genomic Data
Source: G3 (Bethesda). 2015 Oct 4;5(12):2629–37. doi: 10.1534/g3.115.019513 (PMC4683636; doi:10.1534/g3.115.019513)
Supplement: Supporting Information [file supp_g3.115.019513_FileS1.docx]

File S1: Variance components estimated using the model MAD transformed to the breeding model as proposed by Vitezica et al. (2013)^*^

| **Population** | **Trait** | **Variance components** | | | |  | **Variance explained** | |
| --- | --- | --- | --- | --- | --- | --- | --- | --- |
|  |  | $\sigma_{e}^{2}$ | $\sigma_{L}^{2}$ | $\sigma_{\mathbf{Aa}}^{2}$ | $\sigma_{\mathbf{Dd}}^{2}$ | **** | $\sigma_{\mathbf{Aa}}^{2}$/$\sigma_{\mathbf{P}}^{2}$ | $\sigma_{\mathbf{Dd}}^{2}$/$\sigma_{\mathbf{P}}^{2}$ |
| Landrace | NT | 0.714 ± 0.046 |  | 0.392 ± 0.063 | 0.032 ± 0.026 |  | 0.344 ± 0.045 | 0.028 ± 0.023 |
|  | BF | 1.081 ± 0.137 | 0.362 ± 0.118 | 1.558 ± 0.197 | 0.251 ± 0.121 |  | 0.478 ± 0.045 | 0.077 ± 0.037 |
|  | DG | 1,211 ± 141 | 315 ± 104 | 692 ± 143 | 328 ± 136 |  | 0.271 ± 0.049 | 0.129 ± 0.052 |
|  | NT | 0.576 ± 0.041 |  | 0.333 ± 0.046 | 0.069 ± 0.031 |  | 0.340 ± 0.037 | 0.070 ± 0.031 |
| Large White | BF | 0.924 ± 0.106 | 0.307 ± 0.085 | 0.992 ± 0.128 | 0.233 ± 0.094 |  | 0.403 ± 0.041 | 0.095 ± 0.038 |
|  | DG | 1,717 ± 143 | 233 ± 109 | 697 ± 127 | 220 ± 117 |  | 0.243 ± 0.040 | 0.077 ± 0.040 |
| Pietrain | BF | \| 0.567 ± 0.056 \| \| --- \| | \| 0.083 ± 0.039 \| \| --- \| | \| 0.495 ± 0.075 \| \| --- \| | \| 0.066 ± 0.046 \| \| --- \| |  | \| 0.407 ± 0.049 \| \| --- \| | \| 0.055 ± 0.038 \| \| --- \| |
|  | DG | 1,492 ± 163 | 151 ± 103 | 945 ± 169 | 427 ± 150 |  | 0.312 ± 0.048 | 0.141 ± 0.048 |

^*^Vitezica *et al.* (2013), *Genetics* 195 (4):1223-1230.
